# The combination of a neprilysin inhibitor (sacubitril) and angiotensin-II receptor blocker (valsartan) attenuates glomerular and tubular injury in the Zucker Obese rat

**DOI:** 10.1186/s12933-019-0847-8

**Published:** 2019-03-25

**Authors:** Javad Habibi, Annayya R. Aroor, Nitin A. Das, Camila M. Manrique-Acevedo, Megan S. Johnson, Melvin R. Hayden, Ravi Nistala, Charles Wiedmeyer, Bysani Chandrasekar, Vincent G. DeMarco

**Affiliations:** 1 Diabetes and Cardiovascular Center, University of Missouri School of Medicine, Columbia, MO USA; 2 Division of Endocrinology and Metabolism, Department of Medicine, University of Missouri-Columbia School of Medicine, D110, DC043.0, One Hospital Dr, Columbia, MO 65212 USA; 3 Research Service, Harry S. Truman Memorial Veterans Hospital, Columbia, MO USA; 4 Cardiothoracic Surgery, University of Texas Health Science Center, San Antonio, TX USA; 5 Division of Nephrology, Department of Medicine, University of Missouri, Columbia, MO USA; 6 College of Veterinary Medicine, University of Missouri, Columbia, MO USA; 7 Division of Cardiology, Department of Medicine, University of Missour, Columbia, MO USA; 8 Department of Medical Pharmacology and Physiology, University of Missouri, Columbia, MO USA; 9 Department of Obstetrics, Gynecology and Women’s Health, University of Missouri, Columbia, MO USA

**Keywords:** Obesity, Diabetes, Glomerular injury, Tubular injury, Neprilysin inhibition

## Abstract

**Objective:**

Diabetic nephropathy (DN) is characterized by glomerular and tubulointerstitial injury, proteinuria and remodeling. Here we examined whether the combination of an inhibitor of neprilysin (sacubitril), a natriuretic peptide-degrading enzyme, and an angiotensin II type 1 receptor blocker (valsartan), suppresses renal injury in a pre-clinical model of early DN more effectively than valsartan monotherapy.

**Methods:**

Sixty-four male Zucker Obese rats (ZO) at 16 weeks of age were distributed into 4 different groups: Group 1: saline control (ZOC); Group 2: sacubitril/valsartan (sac/val) (68 mg kg^−1^ day^−1^; ZOSV); and Group 3: valsartan (val) (31 mg kg^−1^ day^−1^; ZOV). Group 4 received hydralazine, an anti-hypertensive drug (30 mg kg^−1^ day^−1^, ZOH). Six Zucker Lean (ZL) rats received saline (Group 5) and served as lean controls (ZLC). Drugs were administered daily for 10 weeks by oral gavage.

**Results:**

Mean arterial pressure (MAP) increased in ZOC (+ 28%), but not in ZOSV (− 4.2%), ZOV (− 3.9%) or ZOH (− 3.7%), during the 10 week-study period. ZOC were mildly hyperglycemic, hyperinsulinemic and hypercholesterolemic. ZOC exhibited proteinuria, hyperfiltration, elevated renal resistivity index (RRI), glomerular mesangial expansion and podocyte foot process flattening and effacement, reduced nephrin and podocin expression, tubulointerstitial and periarterial fibrosis, increased NOX2, NOX4 and AT_1_R expression, glomerular and tubular nitroso-oxidative stress, with associated increases in urinary markers of tubular injury. None of the drugs reduced fasting glucose or HbA1c. Hypercholesterolemia was reduced in ZOSV (− 43%) and ZOV (− 34%) (p < 0.05), but not in ZOH (− 13%) (ZOSV > ZOV > ZOH). Proteinuria was ameliorated in ZOSV (− 47%; p < 0.05) and ZOV (− 30%; p > 0.05), but was exacerbated in ZOH (+ 28%; p > 0.05) (ZOSV > ZOV > ZOH). Compared to ZOC, hyperfiltration was improved in ZOSV (p < 0.05 vs ZOC), but not in ZOV or ZOH. None of the drugs improved RRI. Mesangial expansion was reduced by all 3 treatments (ZOV > ZOSV > ZOH). Importantly, sac/val was more effective in improving podocyte and tubular mitochondrial ultrastructure than val or hydralazine (ZOSV > ZOV > ZOH) and this was associated with increases in nephrin and podocin gene expression in ZOSV (p < 0.05), but not ZOV or ZOH. Periarterial and tubulointerstitial fibrosis and nitroso-oxidative stress were reduced in all 3 treatment groups to a similar extent. Of the eight urinary proximal tubule cell injury markers examined, five were elevated in ZOC (p < 0.05). Clusterin and KIM-1 were reduced in ZOSV (p < 0.05), clusterin alone was reduced in ZOV and no markers were reduced in ZOH (ZOSV > ZOV > ZOH).

**Conclusions:**

Compared to val monotherapy, sac/val was more effective in reducing proteinuria, renal ultrastructure and tubular injury in a clinically relevant animal model of early DN. More importantly, these renoprotective effects were independent of improvements in blood pressure, glycemia and nitroso-oxidative stress. These novel findings warrant future clinical investigations designed to test whether sac/val may offer renoprotection in the setting of DN.

## Background

The impact of obesity and diabetes on kidney function and structure is well recognized due to increased incidences of diabetes-associated chronic kidney disease (CKD) and end stage renal disease (ESRD) [1–3]. The progression of CKD is associated with persistent activation of the renin-angiotensin system (RAS), with consequent development of hyperfiltration, proteinuria, glomerular mesangial expansion, and tubulointerstitial fibrosis, resulting ultimately in decreased glomerular filtration rate (GFR) [3, 4]. Moreover, pre-diabetic individuals with impaired glucose tolerance and insulin resistance are at higher risk for developing CKD [5]. The standard of care for the management of kidney injury involves administration of angiotensin-converting enzyme inhibitors (ACEi) or angiotensin II (Ang-II) type 1 receptor (AT_1_R) blockers (ARB) [6–9]. However, in many subjects with obesity and diabetes, there is a relentless progression of CKD despite the use of these inhibitors, suggesting a critical need for developing additional therapeutic strategies beyond ARB and ACEi. In this regard, one of the attractive new targets for treating kidney injury involves neprilysin, an endopeptidase that is positively associated with cardiovascular (CV) morbidity and mortality in HF patients [10]. Neprilysin metabolizes several small vasoactive peptides, including atrial (ANP) and brain (BNP) natriuretic peptides, Ang-II, bradykinin and endothelin-1, among others [11]. Natriuretic peptides (NP) are well known to induce cGMP synthesis that promotes natriuresis, diuresis and vasodilation and have been shown to inhibit mesangial cell proliferation and kidney fibrosis [12–15]. Earlier attempts to reduce the degradation of NPs utilizing an inhibitor of neprilysin alone (NEPi) fell short due to an increase in Ang-II levels that promote sodium retention, vasoconstriction and cardiac fibrosis [11]. Subsequently, NEPi were combined with ACEi; however their combination increased the risk of angioedema due to inhibition of bradykinin degradation, precluding its clinical utility [11].

The next iteration involved combination of NEPi with an ARB so that the latter suppresses the effects of increased Ang-II and the accompanying vasoconstrictor and aldosterone promoting effects. Subsequently, in July 2015, the FDA approved Entresto (LCZ696), a combination of the NEPi, sacubitril (sac), and the ARB, valsartan (val), for treatment of heart failure (New York Heart Association, class II–IV) with reduced (≤ 40%) ejection fraction (HFrEF). Analysis of the PARADIGM-HF (Prospective Comparison of ARNI with ACE inhibition to Determine Impact on Global Mortality and Morbidity in Heart Failure) trial, demonstrated that Entresto reduced hospitalization for HF and death from CV causes, compared to enalapril [16–21].

Much recent interest has begun to focus on potential benefits of Entresto on kidney function. In this regard, a recent post hoc analysis of PARADIGM-HF reported that Entresto slowed the rate of decrease in estimated glomerular filtration rate (eGFR) and exhibited favorable effects on CV and kidney outcomes in HFrEF patients with or without CKD, compared to enalapril [22]. More recently, results of the UK HARP-III (United Kingdom Heart and Renal Protection-III) trial, designed to examine the effects of Entresto on kidney function and cardiac biomarkers in patients with moderate to severe CKD, reported comparable renoprotection with Entresto and irbesartan [23]. Another recent secondary analysis of the PARADIGM-HF trial investigating the course of renal disease in patients with type 2 diabetes mellitus (T2DM) indicated that Entresto significantly slowed the rate of decline in eGFR compared to enalapril and this positive result could not be explained by improvement in glycemia or the effect of the drug on the clinical course of HFrEF [24]. However, little is known regarding the underlying mechanisms of sac/val on glomerular and tubular injury, as well as renal microvascular dysfunction, in the setting of obesity and diabetes with early DN, conditions often associated with diastolic dysfunction or heart failure with preserved ejection fraction (HFpEF).

In this study, we examined the effects of sac/val in a model of early stage DN characterized by moderate glomerular and tubular injury [25]. We utilized the Zucker Obese (ZO) rat that displays hyperphagia-induced obesity, brought on by the absence of the leptin receptor, as well as, renal hyperfiltration, hypertension, dyslipidemia, oxidative stress and proteinuria [26–29]. Additionally, the ZO rat is a well characterized model of cardiac diastolic dysfunction and aortic stiffness [30–33]. We hypothesized that sac/val imparts greater kidney functional and structural protections compared to either val monotherapy or hydralazine, the latter being an antihypertensive medication (blood pressure control group) that does not specifically target the RAS. Indeed, supporting our hypothesis, sac/val more effectively suppressed both glomerular and tubular injury compared to val or hydralazine.

## Methods

### Animals

Sixty-four male Zucker Obese (ZO) and six age-matched Zucker Lean (ZL) rats were purchased from Charles River, Inc and housed in a 12 h light/dark cycled room. Animals were cared for in accordance with the National Institutes of Health guidelines. All procedures were approved and performed in accordance with Subcommittee for Animal Safety of the Harry S Truman Veterans Administration and the Institutional Animal Care and Use Committee of the University of Missouri. All ZO rats were weighed prior to the start of the experiment and distributed into four treatment groups so that each group had a similar mean body weight. Beginning at 16 weeks of age, ZO rats received either sac/val (ZOSV) (68 mg kg^−1^ day^−1^), valsartan (ZOV) (31 mg kg^−1^ day^−1^), hydralazine (ZOH) (30 mg kg^−1^ day^−1^) or saline (ZOC) once daily for 10 weeks by oral gavage. Rats were gavaged at the same time each morning (6:00–7:00 a.m. central standard time). Body weights were measured every week thereafter until the end of the experiment (26 weeks of age). Untreated age-matched male ZL rats served as lean controls (ZLC). Six rats were removed from the study due to complications associated with oral gavage.

### Telemetric blood pressure monitoring

We previously reported elevated ambulatory mean arterial pressure (MAP) in ZO rats assessed by radiotelemetric monitoring [30]. Under isoflurane anesthesia (2% isoflurane in a stream of O_2_), a subset of 13 week-old ZO rats (n = 16) were implanted with an abdominal aorta catheter attached to a radio transmitter (TA11PA-C40; Data Sciences International, St. Paul, Minnesota), as previously described [30]. After a 3-week recovery, MAP was monitored in 300-s bins every 15 min for two 12-h light and two 12-h dark cycles (sampling rate, 1000 Hz), and telemetry data were analyzed post hoc. Monitoring periods ended 2 days prior to and approximately 3, 5, 7 and 9 weeks after treatment began. One rat was removed from the study prior to the start of BP monitoring due to complications from transmitter implantation surgery.

### Ultrasound assessment of renal vascular function

We and others have recently utilized a non-invasive Doppler ultrasound procedure to evaluate the RRI in mice, an index of renal microvascular stiffening [34–37]. We further reported an association between increased RRI and microalbuminuria in db/db mice, a model of more advanced DN than ZO rats [34]. One to 2 days prior to sacrifice, B-mode and Pulsed-Wave Doppler ultrasound were performed by a single experienced observer in a blinded fashion using a Vevo 2100 (FUJIFILM VisualSonics, Ontario, Canada) for measurement of RRI. RRI is considered to be a predictor of renal vascular resistance [38]. Images of the left renal artery were acquired in Color Doppler mode to visualize arterial blood flow. A sample volume was placed in the left renal artery just proximal to the kidney pelvis and PW spectra were captured and analyzed offline to acquire peak systolic velocity (PSV) and lowest diastolic velocity (LDV) determined from the velocity time integral. Parameters were measured in triplicate using three separate spectra. RRI was calculated as a ratio of (PSV–LDV) to PSV.

### Urine collection and chemistry

Three to four days prior to the end of the study, rats were placed in metabolic chambers for 24 h urine collection, with full access to food and water. Immediately following urine collection, urinary albumin and creatinine were measured using a Sieman’s DCA Vantage Analyzer per manufacturer’s instructions. Subsequently, frozen urine samples (stored at − 80 °C) were thawed on ice and analyzed for a number of glomerular and tubular injury markers. Protein and creatinine (enzymatic) concentrations in urine were analyzed on an automated clinical chemistry analyzer (Beckman-Coulter AU680, Beckman-Coulter, Brea, CA) using commercially available assays. A total of ten urinary injury markers were quantified; *N*-acetyl-β-d-glucosaminidase (β-NAG) and γ-glutamyl transpeptidase (GGT) were determined by colorimetric assay (Roche Diagnostics, Indianapolis, IN), calbindin, clusterin, glutathione-S-transferase-α (GSTα), interferon-γ-inducible protein-10 (IP-10), kidney injury molecule-1 (KIM-1), osteopontin (OPN), tissue inhibitor of metalloprotease (TIMP-1) and vascular endothelial growth factor (VEGF) were measured using the MILLIPLEX MAP Rat Kidney Magnetic Bead Panel 1-Toxicity Multiplex Assay (RKTX1MAG-37K, Millipore/Sigma) on the Luminex xMAP platform following manufacturer’s instructions. The levels of GSTα and OPN were below detection limits and are not reported.

### Blood collection and chemistry

Ten minutes prior to euthanasia, blood was collected from fasted (5 h fast) conscious rats by venipuncture of the tail vein for measures of fasting glucose and insulin. Blood glucose was measured immediately with a glucometer (AlphaTRAK, Abbott). Plasma was separated from the remaining blood sample and stored at − 80 °C for later analysis of insulin. Insulin was quantified by a commercial laboratory using a rat-specific ELISA (Comparative Clinical Pathology Services, Columbia, MO). Insulin resistance was assessed by homeostatic model assessment of insulin resistance (HOMA-IR) (Table 1). Animals were euthanized by exsanguination under anesthesia (4–5% isoflurane) and the large blood sample collected by cardiac puncture was processed to plasma and stored at − 80 °C for later measurement of creatinine, total cholesterol, triglycerides, sodium, aspartate aminotransferase (AST), and alanine aminotransferase (ALT) using an automated clinical chemistry platform (Beckman-Coulter AU680, Beckman-Coulter, Brea, CA) and commercially available assays (Beckman-Coulter, Brea, CA). Plasma cystatin c, a biomarker of GFR, was determined using a species-specific ELISA according to manufacturer’s specifications (RayBiotech, Norcross, GA).

**Table 1:** Phenotypic parameters of Lean and Obese Zucker rats at the end of the 10 treatment period

Parameter (n)	ZLC (6)	ZOC (7–13)	ZOSV (7–13)	ZOV (7–13)	ZOH (7–14)
Body weight (g)	472 ± 17	752 ± 17*^§^	709 ± 27*	733 ± 16*	657 ± 28*^†^
Left kidney weight/TL (mg/mm)	28.7 ± 0.8	52.9 ± 1.8^a^	54.6 ± 3.9*	52.3 ± 2.1	65.9 ± 9.6*
Epididymal fat (g)	7.4 ± 0.9	20.6 ± 1.5*	17.9 ± 1.3*	21.1 ± 1.2*	16.6 ± 1.4*
Retroperitoneal fat (g)	7.2 ± 0.8	46.6 ± 2.8*	48.0 ± 3.4*	50.4 ± 3.6*^§^	36.6 ± 2.7*
Blood parameters					
HbA1c (%)	3.7 ± 0.1	5.0 ± 0.5	4.4 ± 0.3	5.5 ± 0.5^a^	4.6 ± 0.4
Fasting glucose (mg dL^−1^) (mmol L^−1^)	100 ± 5	148 ± 7^a^	169 ± 25	184 ± 17^a^	174 ± 25
Fasting insulin mU L^−1^	25.7 ± 10	109 ± 19*	113 ± 32*	105 ± 15*	119 ± 23*
HOMA-IR (FG FI)/405	7 ± 3	42 ± 10*	45 ± 9*	53 ± 11*	45 ± 11*
Cholesterol (mg dL^−1^)	100 ± 11	468 ± 66*	265 ± 30^†§^	308 ± 35*^†^	407 ± 69*
Triglycerides (mg dL^−1^)	84 ± 12	2000 ± 394*	1348 ± 307*	1259 ± 214*	2550 ± 719*
Creatinine (mg dL^−1^)	0.32 ± 0.02	0.45 ± 0.09	0.29 ± 0.04	0.33 ± 0.04	0.44 ± 0.13
AspAT (U L^−1^)	78 ± 14	240 ± 61	202 ± 43	248 ± 59	127 ± 22
AlaAT (U L^−1^)	40 ± 5	139 ± 33*	132 ± 30	161 ± 30*	73 ± 12
Sodium (mEq L^−1^)	138 ± 1	133 ± 2	135 ± 1	134 ± 1	127 ± 4

ZOC, ZO control; ZOSV, ZO treated with Sac/val; ZOV, ZO treated with valsartan; ZOH, ZO treated with hydralazine; TL, tibia length; HOMA-IR, homeostatic model assessment of insulin resistance; AspAT, aspartate aminotransferase; AlaAT, alanine aminotransferase

Values are mean ± SE and sample sizes are shown in parentheses. ANOVA post hoc comparisons: * P < 0.05 vs ZLC; ^†^ P < 0.05 versus ZOC; ^§^ P < 0.05 versus ZOH. Two-tailed t-tests: ^a^ P < 0.05 versus ZLC; ^b^ P < 0.05 versus ZOC

### Quantification of tubulointerstitial and periarterial fibrosis

Two mm-thick slices of kidney were fixed in paraformaldehyde, embedded in paraffin, sectioned at five microns and stained for collagens using picro-Sirius-red (PSR), as previously described [34, 39]. For each animal an average estimate of interstitial fibrosis was calculated from four randomly selected regions. Periarterial fibrosis was determined by normalizing the area of PSR stain surrounding an arteriole to arteriolar diameter (diameter = circumference of artery/3.14). Average values for each animal were based on measurements made on four randomly selected renal arterioles from ZLC (n = 6), ZOC (n = 5), ZOSV (n = 5), ZOV (n = 6) and ZOH (n = 7).

### Quantification of intraglomerular mesangial expansion

Five micrometer sections of paraffin embedded kidney were dewaxed, rehydrated and stained with periodic acid-Schiff (PAS) stain that detects glomerular mesangial matrix. Briefly, three randomly selected 40× images (1392 pixels wide × 1040 pixels high) containing cortical glomeruli were captured for each kidney using MetaVue software. Subsequently, the images were leveled using Photoshop; then all colors except the hot pink color, which stains the glomerular mesangium, were filtered. In each of these modified images we quantified the amount of pink stain in a 500 × 500 pixel area within a glomerular tuft and expressed as Gray Scale Intensity. For each animal an average estimate of the relative quantity of mesangium was calculated from triplicate determinations of gray scale intensity of pink coloration. Samples from five to seven rats from each of the five treatment groups were analyzed.

### Ultrastructure analysis with transmission electron microscopy

Details of kidney cortical tissue preparation, sectioning, staining and viewing are as previously described [26]. A JOEL JEM 1400 transmission electron microscope was utilized to review three fields randomly chosen per rat to obtain three 2000× images of the glomerulus and proximal tubules. Tissues from three different animals in each group were processed and examined with TEM for the presence of ultrastructural lesions, and representative images are displayed in figures.

### Glomerular and tubular 3-nitrotyrosine (3-NTY)

We evaluated the levels of 3-NTY as a marker of kidney nitrosylated oxidation products caused by formation of peroxynitrite. Five micrometer sections of the kidney were initially quenched of endogenous peroxidase and incubated with 1:200 rabbit polyclonal anti-3-NTY antibody overnight (Chemicon, Temecula, CA) [40, 41]. Sections were washed and incubated with appropriate secondary antibody and signals visualized by diaminobenzidine (DAB) chromogen system (DAKO, Carpinteria, CA). Using a 50i Nikon microscope, five randomly selected 10× bright-field images from each section were captured with a CoolSNAP cf camera. Signal intensities of brownish color, which is indicative of the 3-NTY level, were quantified by MetaVue software.

### mRNA expression by quantitative real time PCR (RT-qPCR)

DNA-free total RNA was prepared using the RNAqueous^®^-4PCR kit (Ambion). RNA quality was assessed by capillary electrophoresis using the Agilent 2100 Bioanalyzer (Agilent Technologies, Palo Alto, CA). All RNA samples used for quantitative PCR had RNA integrity numbers greater than 9.0 (scale = 1–10) as assigned by default parameters of the Expert 2100 Bioanalyzer software package (v2.02). mRNA expression was analyzed by RT-qPCR using best coverage TaqMan™ probes from Thermo Fisher Scientific-Applied Biosystems (Nox2 or Cybb, Rn00576710_m1; Nox4, Rn00585380_m1; AT_1_R or Agtr1a, Rn02758772_s1; Nephrin or NPHS1, Rn00674268_m1; Podocin or NPHS2, Rn00709834_m1; Collagen Iα1 or Col1α1, Rn01463848_m1; Fibronectin or FN1, Rn00569575_m1) [42–44]. 18S served as the internal control. No template controls were also performed for each assay, and samples processed without the reverse transcriptase step served as negative controls. Each cDNA sample was run in triplicate, and the amplification efficiencies of all primer pairs were determined by serial dilutions of input template. Gene expression is presented as a ratio of specific target to 18S mRNA and expressed as a fold change from baseline in ZLC group (n = 4 per group). Data were analyzed using the 2^−ΔΔCt^ method.

### Statistical analysis

Results are reported as the mean ± SE. One way ANOVA and post hoc t-tests (Fisher’s LSD), or corresponding non-parametric Kruskal–Wallis (Dunn’s), as indicated, were performed to examine differences in outcomes between ZL rats and control and treated ZO groups. Alternatively, we performed two-tailed Student t-tests between two groups when ANOVA post hoc tests indicated p = 0.10. A p value < 0.05 was considered significant. Differences in MAP at the last measurement period following administration of either sac/val, val or hydralazine versus untreated ZO rats were determined by Student t-tests. Sample sizes are listed in tables and figures.

## Results

### Plasma biochemical parameters and glycemic control

Compared to ZLC, ZOC exhibited increased body weight, epididymal and retroperitoneal fat pad masses, fasting glucose, fasting insulin and insulin resistance (assessed by HOMA-IR) (p < 0.05) (Table 1). ZOC tended to have elevated HbA1c, but the increase relative to ZLC did not reach statistical significance. These parameters were largely unaffected by sac/val, val or hydralazine. Nonetheless, it is noteworthy that ZOV had the highest HbA1c, fasting glucose and insulin resistance of the five groups examined. In concert with dysglycemia, ZOC exhibited hyperlipidemia indicated by increased fasting cholesterol and triglycerides (Table 1). Compared to ZOC, cholesterol was significantly lower in ZOSV (− 43%) and ZOV (− 34%) (p < 0.05%). Triglycerides also tended to be lower in these two groups; however, hydralazine failed to lower these plasma lipids compared to ZOC. The liver injury markers, AST and ALT tended to be elevated in all ZO groups. There were no significant differences in plasma sodium levels between ZL and ZO rats (p < 0.05).

### Sacubitril/valsartan prevents progression of hypertension in the ZO rat

**Fig. 1:**
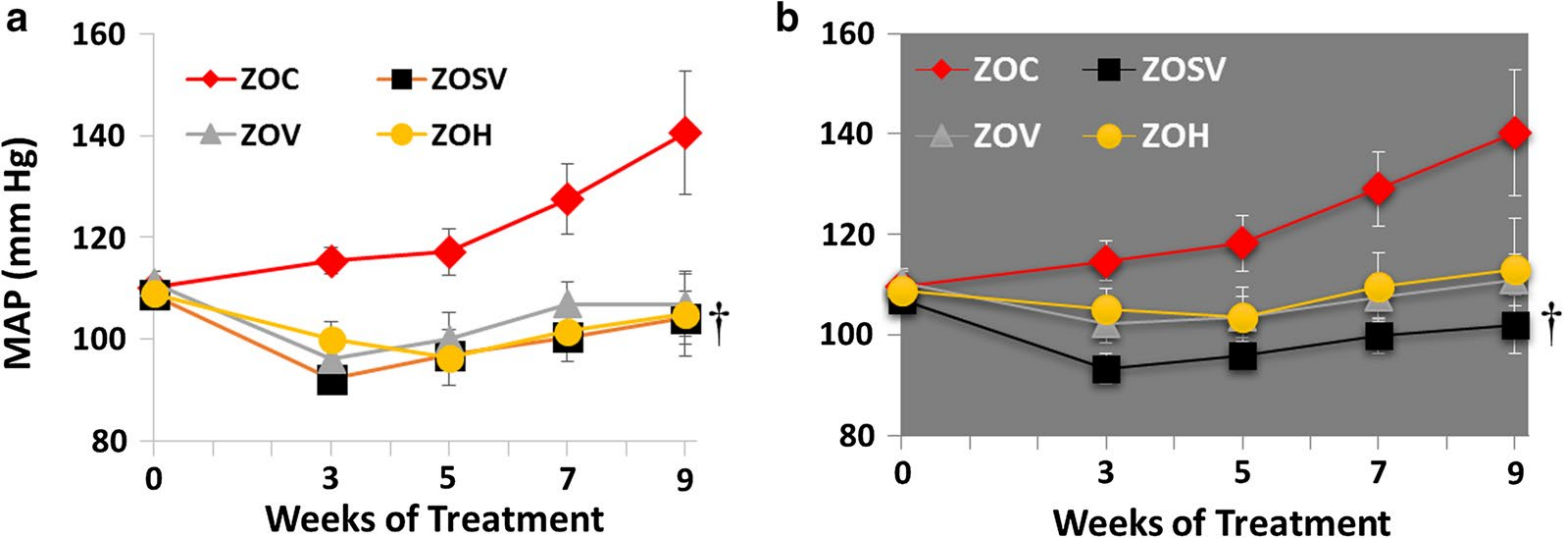
Ambulatory blood pressure was monitored periodically utilizing radio-telemetric transmitters. Mean arterial pressure (MAP) was recorded prior to the beginning of treatment and after 3, 5, 7 and 9 weeks of treatment during the **a** light and **b** dark cycles. Compared to ZLC, MAP increased throughout the course of treatment in ZOC. After 9 weeks of treatment MAP was significantly reduced in ZOSV during the light and dark periods compared to ZOC (p < 0.05 indicated by the dagger symbol). n = 4, 4, 4, 3 and 4 for ZLC, ZOC, ZOSV, ZOV and ZOH, respectively

Baseline (i.e., pre-treatment) MAP, during both the light and the dark periods, was approximately 110 mmHg (Fig. 1). Near the end of the study, MAP increased by 28% to 141 ± 12 mmHg during both periods in ZOC. Relative to baseline MAP, treatment with sac/val, val or hydralazine tended to lower MAP by 4.2% in ZOSV, 3.9% in ZOV and 3.7% in ZOH, however, only ZOSV reached statistical significance by the end of the study during both light and dark cycles (p < 0.05).

### Sacubitril/valsartan improves glomerulopathy in the ZO rat

The ratio of urine protein to enzymatic creatinine reflecting proteinuria is routinely used as a marker of kidney injury in ZO rats [27, 32, 40]. In the present study, ZOC exhibited a nearly threefold increase in the protein to creatinine ratio compared to ZLC (p < 0.05; Table 2). Sac/val reduced proteinuria by 47% (Students *t* test; p < 0.05 versus ZOC) compared to a 31% reduction by val (p > 0.05 versus ZOC). On the other hand, hydralazine tended to increase proteinuria compared to ZOC (+ 28%; p > 0.05), ZOSV (+ 140%; p < 0.5) and ZOV (+ 84%; p < 0.05) (Table 2). Differences in the amount of protein excreted per day were consistent with differences in proteinuria among groups. Protein excretion was over fourfold higher in ZOC compared to ZLC (p < 0.05, Table 2). Compared to ZOC, sac/val and val reduced protein excretion by 49 and 24%, respectively (p < 0.05). On the other hand, hydralazine tended to increase protein excretion compared to ZOC (+ 23%; p > 0.05), ZOSV (+ 141%; p < 0.5) and ZOV (+ 62%; p < 0.5). Although there were no significant differences in the urine albumin to creatine ratio among groups, the trends among the groups were consistent with those of proteinuria (Table 1).

**Table 2:** Urine parameters of Zucker Lean Control (ZLC) and Zucker Obese (ZO) rats after 9 weeks of treatment

Parameter	ZLC (6)	ZOC (10)	ZOSV (10)	ZOV (10)	ZOH (9)
Proteinuria (mg mgCr^−1^)	4.3 ± 1.4	16.7 ± 3.0*	8.9 ± 1.5^ab§^	11.6 ± 2.8^§^	21.4 ± 4.2*
Creatinine (mg dL^−1^)	335 ± 65	174 ± 16*	184 ± 20*	167 ± 16*	148 ± 13*
Protein (mg dL^−1^)	1061 ± 314	2808 ± 446*	1632 ± 304^†§^	1889 ± 392	2986 ± 510*
Urine volume (mL)	7.7 ± 0.9	17.0 ± 3.5	16.8 ± 3.4	17.9 ± 2.9	17.4 ± 2.1
Protein excretion (mg day^−1^)	91 ± 0	396 ± 6*	203 ± 59^†§^	301 ± 62^§^	489 ± 83*
Albuminuria (mg gCr^−1^)	29.9 ± 8.6	43.2 ± 6.3	33.6 ± 7.5	46.1 ± 6.8	62.7 ± 13.4
Sodium excretion (mmol day^−1^ g BW^−1^)	1.37 ± 0.16	2.04 ± 0.41	2.11 ± 0.46	2.40 ± 0.34^a^	2.67 ± 0.20^a^
β-NAG (U mgCr^−1^)	0.020 ± 0.002	0.042 ± 0.007*	0.038 ± 0.004^a^	0.038 ± 0.003^a^	0.061 ± 0.008*
GGT (U mgCr^−1^)	0.05 ± 0.1	0.31 ± 0.1^a^	0.27 ± 0.1	0.23 ± 0.1	0.21 ± 0.2
IP-10 (pg mgCr^−1^)	0.85 ± 0.11	1.26 ± 0.22	1.35 ± 0.15	1.10 ± 0.20	1.06 ± 0.15
Calbindin (ng mgCr^−1^)	0.25 ± 0.03	0.35 ± 0.07	0.29 ± 0.07	0.38 ± 0.06	0.26 ± 0.04
Clusterin (ng mgCr^−1^)	1.54 ± 0.24	4.70 ± 0.49*	3.15 ± 0.37*^†^	3.62 ± 0.34*^†^	3.74 ± 0.27*
KIM-1 (ng mgCr^−1^)	0.006 ± 0.002	0.021 ± 0.003*	0.013 ± 0.002^b§^	0.015 ± 0.003*	0.021 ± 0.004*
TIMP-1 (ng mgCr^−1^)	0.16 ± 0.08	0.57 ± 0.15^a^	0.36 ± 0.10	0.37 ± 0.08	0.43 ± 0.12
VEGF (ng mgCr^−1^)	0.010 ± 0.00	0.015 ± 0.00	0.012 ± 0.00	0.015 ± 0.00	0.011 ± 0.00

ZOC, ZO control; ZOSV, ZO treated with Sac/val; ZOV, ZO treated with valsartan; ZOH, ZO treated with hydralazine; β-NAG, *N*-acetyl-β-glucosaminadase; GGT, γ-glutamyl transferase; IP-10, interferon gamma (IFN-γ)-inducible protein; KIM-1, kidney injury molecule-1; TIMP-1, tissue inhibitor of metalloproteinase-1; VEGF, vascular endothelial growth factor

Sac/val (ZOSV) prevents proteinuria and improves select urine markers of kidney injury, including clusterin and KIM-1. Values are mean ± SE, n = 6–10 (sample sizes shown in parentheses). ANOVA post hoc comparisons: * P < 0.05 versus ZLC; ^†^ P < 0.05 versus ZOC; ^§^ P < 0.05 versus ZOH. Two-tailed t-tests; ^a^ P < 0.05 versus ZLC; ^b^ P < 0.05 versus ZOC

**Fig. 2:**
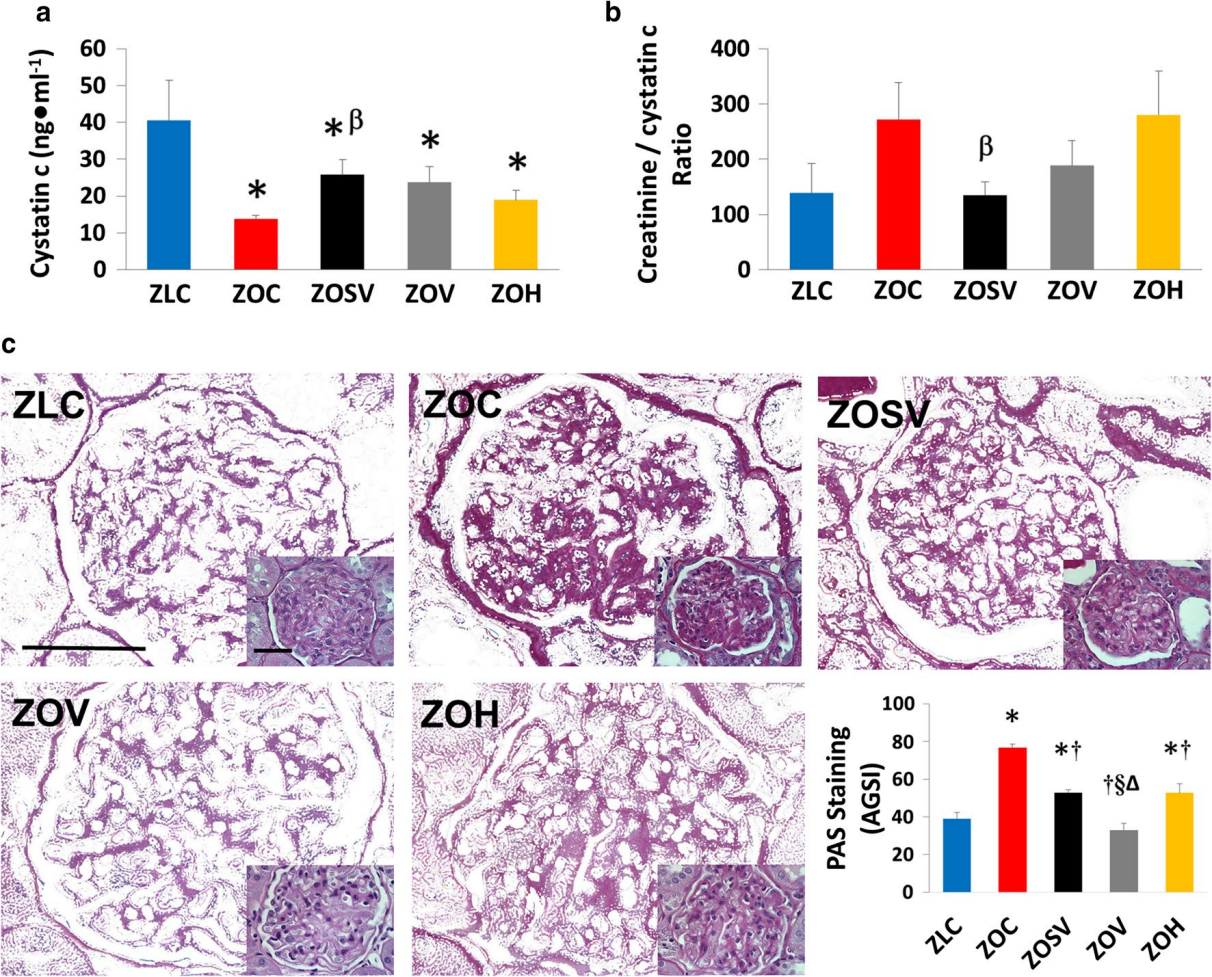
Sacubitril/valsartan improves filtration barrier injury in the Zucker Obese (ZO) rat. **a** Measures of plasma cystatin c, a surrogate marker of glomerular filtration rate. **b** Ratio of plasma creatinine to cystatin c, which is inversely associated with kidney injury. n = 6–9 for each treatment group. **c** Mesangial expansion is reduced by combination and monotherapies. Representative micrographs of PAS-stained cortical glomeruli. All colors except hot pink PAS stain were filtered. The small insets in the lower right corner of each representative micrograph show the original raw unfiltered PAS-stained images. Scale bars equal 50 μm. n = 5 for each treatment group, except for hydralazine (n = 7). Bar graphs show quantitative analysis of PAS stain in the glomerular mesangium. Symbols: *p < 0.05 versus ZLC; ^†^p < 0.05 versus ZOC; ^Δ^p < 0.05 versus ZOSV; ^§^p < 0.05 versus ZOH; ^α^indicates p < 0.05 vs ZLC by two tailed T-test; ^β^p < 0.05 versus ZOC by two-tailed T-test

Compared to ZLC, plasma cystatin c, a biomarker of GFR, was significantly lower in ZOC (66%, p < 0.05) (Fig. 2a), indicating significant derangement in GFR and hyperfiltration. On the other hand, plasma cystatin c levels tended to be higher by 86, 71, and 36%, respectively, in ZOSV, ZOV and ZOH, compared to ZOC, suggesting improvement of hyperfiltration. Sac/val treated rats were the only group (ZOSV) that showed a significantly elevated cystatin c level compared to ZOC (t-test, p < 0.05). However, no significant differences were observed among treatment groups in plasma creatinine (Table 1). An increase in the ratio of creatinine to cystatin c is associated with decreased kidney function indicating derangement in GFR [45]. Compared to ZLC, this injury marker tended to be higher in ZOC and ZOH (p > 0.05). Moreover, compared to ZOC, the creatinine to cystatin c ratio was significantly lower in ZOSV (t-test, p < 0.05), but not ZOV. The ratios were similar in ZLC and ZOSV.

Glomerular mesangial expansion, characterized by increased PAS-positive staining of the mesangial matrix, was increased in ZOC, compared to ZLC, and this was partially or fully prevented in all treated groups (ZOSV, ZOV and ZOH) (Fig. 2). Valsartan appears to be more effective in preventing mesangial expansion (ZOV > ZOSV = ZOH; p < 0.05).

**Fig. 3:**
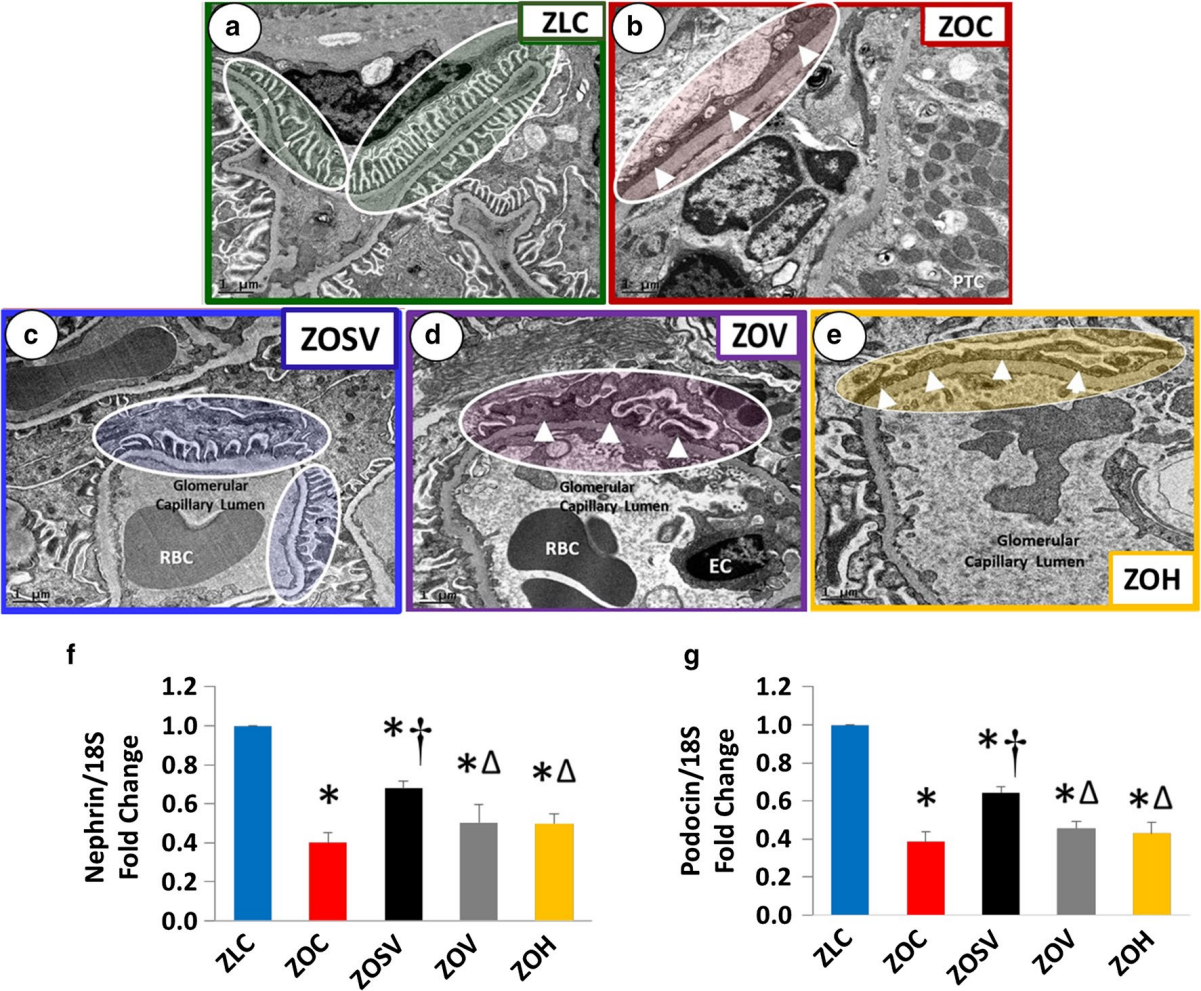
Sacubitril/valsartan improves ultrastructure of the glomerular filtration barrier. **a** Representative micrographs show normal ultrastructure of the glomerular podocyte and adjacent slit pores in ZLC kidneys. **b** ZOC exhibited profound loss of endothelial fenestrae along with podocyte effacement and loss of slit pore diaphragm which were partially restored by sac/val treatment. Podocyte foot processes were markedly effaced and flattened in **b** ZOC, **d** ZOV and **e** ZOH, however they appeared more normal in **c** ZOSV. All images are ×2000 magnification; scale bar = 1 μm. **f**–**g** Suppression of nephrin and podocin gene expression is rescued in ZOSV, but not ZOV or ZOH. Bar graphs show nephrin and podocin mRNA expression normalized to 18S mRNA and expressed as a fold change from baseline in ZLC group (n = 4 per group). *p < 0.05 versus ZLC; ^†^p < 0.05 versus ZOC; ^Δ^p < 0.05 versus ZOSV

TEM revealed normal ultrastructure of the glomerular podocyte and adjacent slit pores in ZLC kidneys (Fig. 3). ZOC exhibited profound loss of endothelial fenestrae along with podocyte flattening and effacement and loss of slit pore diaphragm, which were partially restored by sac/val (ZOSV). Podocyte foot processes were markedly flattened in ZOC, ZOV and ZOH, however they appeared more normal in ZOSV.

Nephrin and podocin, which co-localize in the glomerulus [46], maintain podocytes and their slit pores. Importantly, the expression of nephrin and podocin are markedly suppressed in the diabetic kidney [47–49]. Supporting these studies, the mRNA expression of nephrin and podocin were reduced by 58 and 52% in ZOC kidneys, compared to ZLC (Fig. 3f–g). However, nephrin and podocin transcripts were similarly elevated in ZOSV compared to ZOC, ZOV and ZOH (p < 0.05; ZOSV > ZOV and ZOH > ZOC), suggesting that the combination therapy is most effective in preventing nephrin and podocin loss.

### Sacubitril/valsartan improves tubulopathy in the ZO rat

#### Urine injury markers

**Fig. 4:**
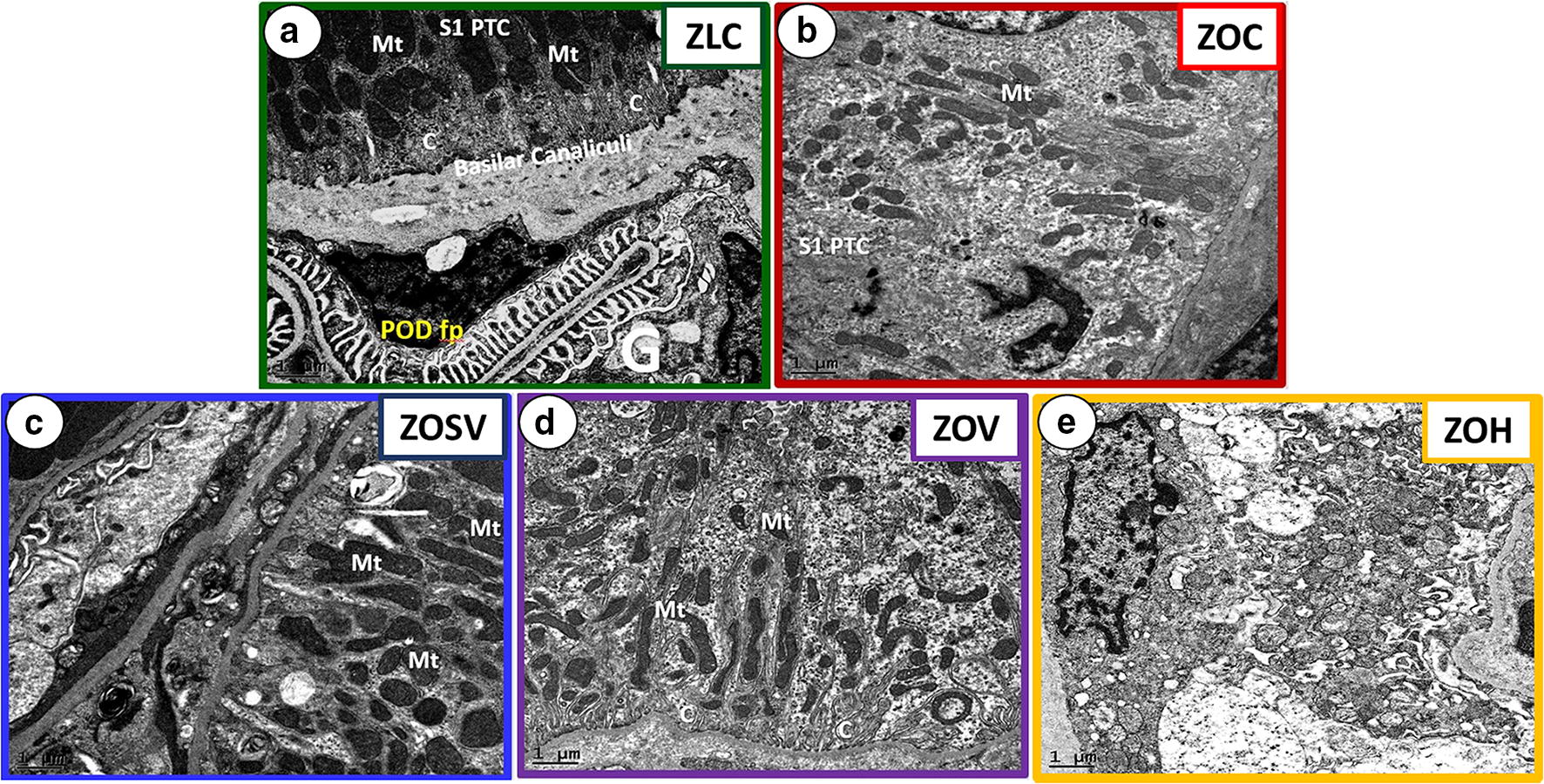
Sacubitril/valsartan improves tubular ultrastructure. Representative TEM images showing ultrastructural remodeling in proximal tubule cells (PTC). **a** Normal basilar S-1 PTC in ZLC showing electron dense mitochondrial and canalicular morphology. Image also shows adjacent glomerulus with normal podocyte foot processes (POD fp). **b** Depicts the loss of basilar mitochondria elongation with fragmentation, as well as loss of canalicular morphology in ZOC. **c** Depicts an improvement in S-1 PTC morphology in ZOSV characterized by elongated mitochondria, but without apparent improvement in canaliculi. **d** Also demonstrates abnormal S-1 PTC morphology in ZOV, however mitochondrial fragmentation is less apparent than in ZOC and some canaliculi appear normal. **e** Depicts a marked increase in mitochondrial fragmentation and abnormal chromatin condensation in the S-1 PTC nucleus in ZOH. These remodeling changes indicate marked cellular degeneration in the PTC. All images are ×2000 magnification; scale bar = 1 μm; G, glomerulus; Mt, mitochondria; C, canaliculi

Five of the eight urinary tubular injury markers quantified were significantly elevated in ZOC, compared to ZLC (Table 2). Of the eight, two tubular injury markers, clusterin and KIM-1, were significantly lower in ZOSV. Clusterin was the only marker that was significantly lower in ZOV compared to ZOC, and its suppression was not as low as in ZOSV. None of these markers were affected by hydralazine (p > 0.05). TEM revealed well-delineated electron dense mitochondrial morphology in ZLC; however ZOC exhibited mitochondrial disorganization with loss of elongation and increased fragmentation (Fig. 4a, b). In ZOSV, mitochondrial organization was improved and the overall ultrastructure pattern was more similar to ZLC (Fig. 4c). The improvement in ZOV was not as marked as in ZOSV (Fig. 4d). Ultrastructural remodeling in ZOH is characterized by increased mitochondrial fragmentation and abnormal chromatin condensation in S1 proximal tubules suggestive of cellular degeneration (Fig. 4e).

#### Interstitial and periarterial fibrosis

**Fig. 5:**
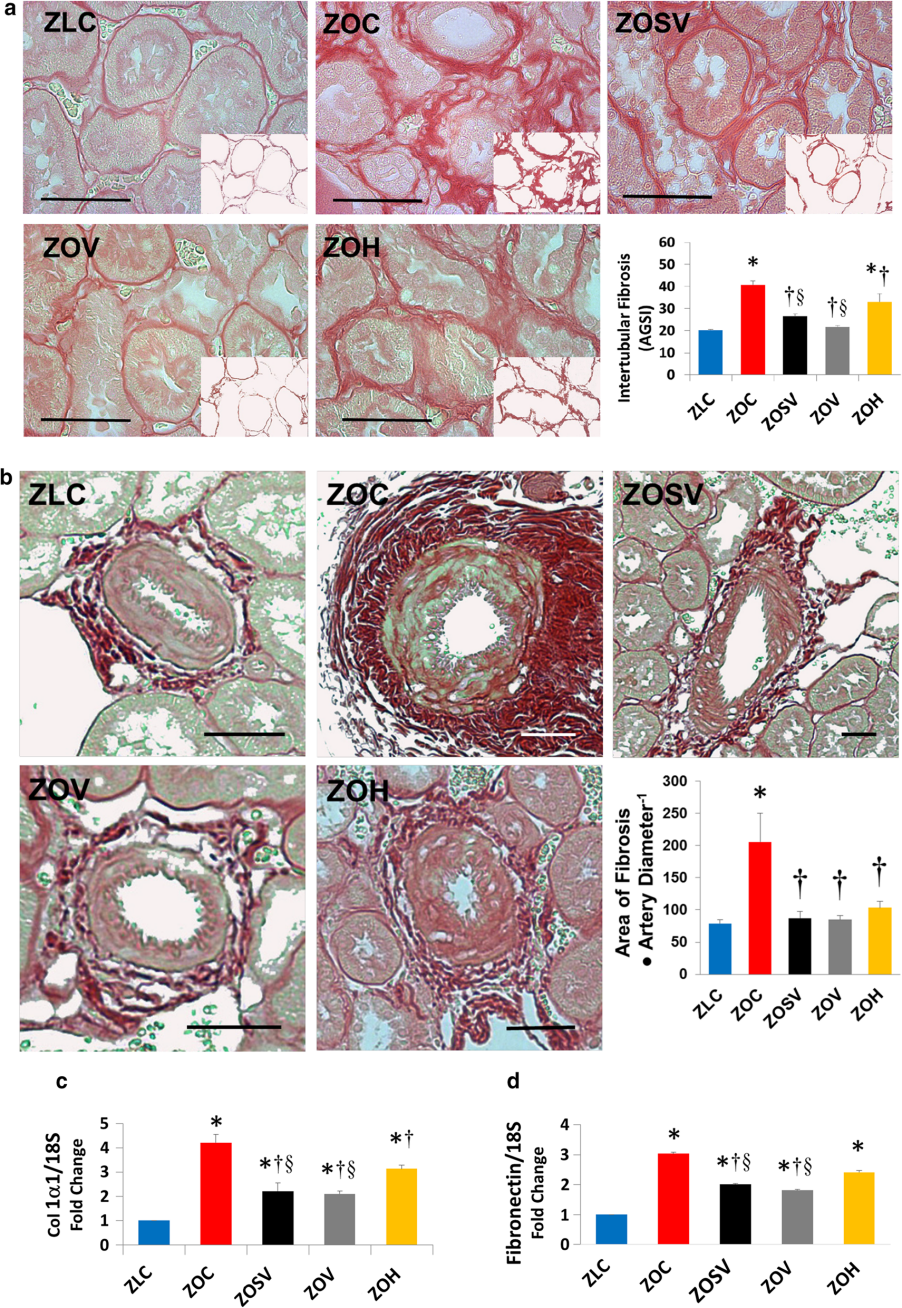
Sacubitril/valsartan reduces **a** tubulointerstitial and **b** periarterial fibrosis in ZO rats. Representative PSR stained images show fibrosis in the renal interstitium in **a** or surrounding an arteriole in **b**. Accompanying bar graphs show quantitative analysis of average intensity of PSR staining in **a** and area of fibrosis normalized to arteriole diameter in **b**. Data are represented by mean ± SE. n = 5–7 rats per group. Symbols: *p < 0.05 versus ZLC; ^†^p < 0.05 versus ZOC; ^§^p < 0.05 versus ZOH. Scale bars = 50 μm. **c**–**e** Bar graphs show Col1α1 and fibronectin mRNA expression normalized to 18S mRNA and expressed as a fold change from baseline in ZLC group (n = 4 per group). *p < 0.05 versus ZLC; ^†^p < 0.05 versus ZOC; ^Δ^p < 0.05 versus ZOSV; ^§^p < 0.05 vs ZOH

Kidney fibrosis in the tubular interstitium, as evaluated by PSR staining, was increased significantly by 203% in ZOC, compared to ZLC (p < 0.05; Fig. 5a). Sac/val (ZOSV), val (ZOV) and hydralazine (ZOH) suppressed interstitial fibrosis by 35, 47 and 19%, respectively (p < 0.05). Further, periarterial fibrosis was increased significantly by 260% in ZOC, compared to ZLC (p < 0.05; Fig. 5b). Sac/val (ZOSV), val (ZOV) and hydralazine (ZOH) suppressed periarterial fibrosis by 58, 59 and 50%, respectively (p < 0.05). Moreover, Col1α1 and fibronectin expression in different treatment groups followed a similar trend as that of PSR staining (Fig. 5c, d).

### Sacubitril/valsartan improves glomerular and tubular oxidative and nitrosative stress

**Fig. 6:**
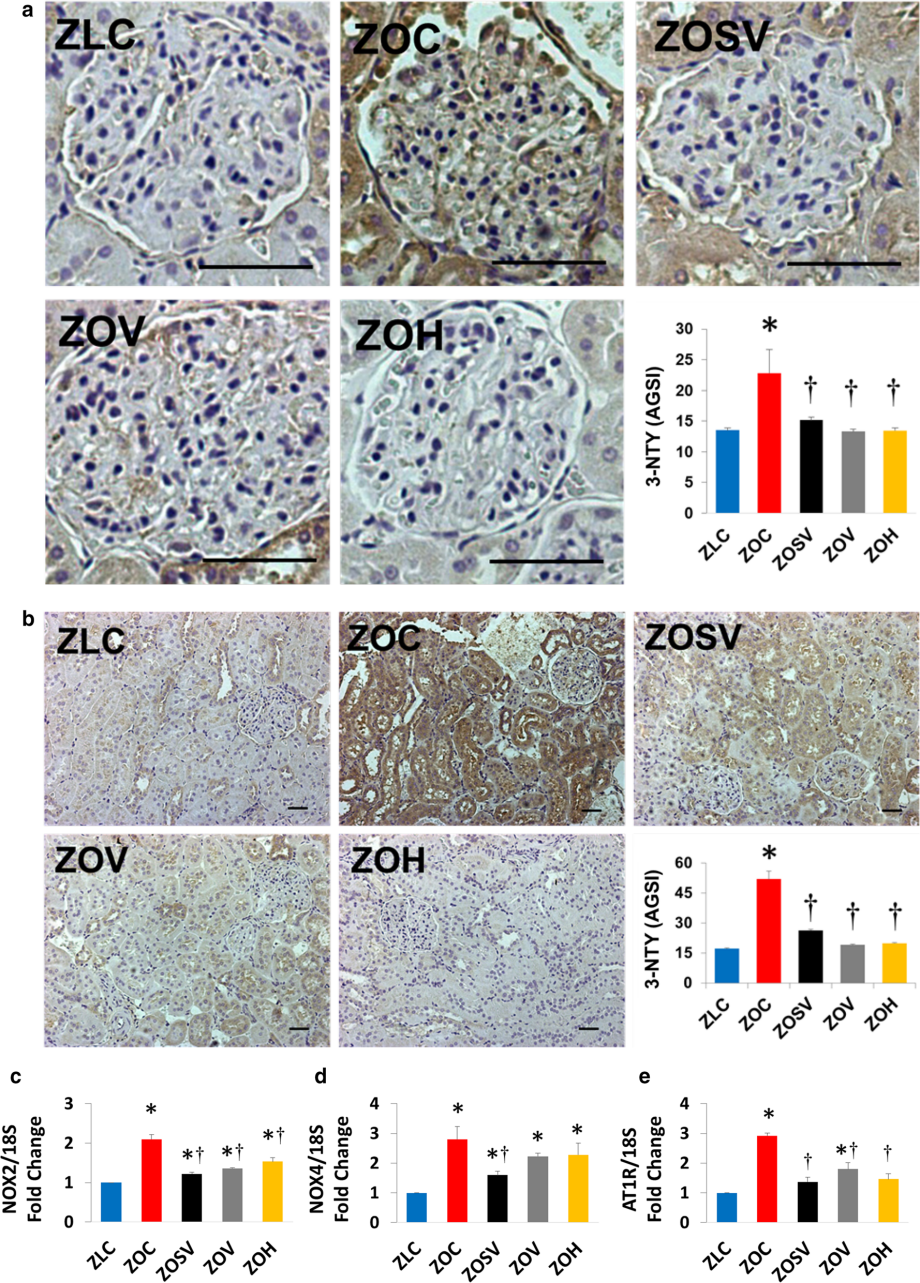
Sacubitril/valsartan reduces **a** glomerular and **b** tubulointerstitial nitroso-oxidative stress in ZO rats. Representative images of 3-nitrotyrosine immunostaining as a marker for nitroso-oxidative stress with accompanying bar graphs showing quantitation of measures of intensity. Data are represented by mean ± SE. n = 5–7 rats per group. **c**–**e** Bar graphs show NOX2, NOX4 and AT_1_R mRNA expression normalized to 18S mRNA and expressed as a fold change from baseline in ZLC group (n = 4 per group). Symbols: *p < 0.05 versus ZLC; ^†^p < 0.05 versus ZOC

Both oxidative and nitrosative stress are implicated in renal injury [27, 34, 50]. Therefore, we analyzed the intensity of 3-NTY staining as a marker of nitroso-oxidative stress in glomeruli and proximal and distal tubules. In glomeruli, 3-NTY staining was increased by 69% in ZOC versus ZLC (p < 0.05), and this increase was inhibited by 34, 42, and 41%, respectively in ZOSV, ZOV and ZOH (p < 0.05; Fig. 6a). The magnitude of increase in 3-NTY in the tubular region was markedly higher in ZOC compared to the relative increase of 3-NTY that was observed in the glomerular region. Specifically, tubular 3-NTY accumulation in ZOC was 304% higher than ZLC and this increase was inhibited by 49, 63 and 62% in ZOSV, ZOV and ZOH, respectively (p < 0.05; Fig. 6b). Since increased NOX2, NOX4 and the AT_1_R expression contributes to oxidative stress in DN [51], we analyzed their expression levels as well. The data for NOX2 and AT_1_R are largely consistent with levels of 3-NTY (Fig. 6c–e). On the other hand, compared to ZOC, the expression of NOX4, which is the predominant NOX isoform in the kidney, was significantly lower in ZOSV, but not ZOV and ZOH (Fig. 6d).

### Sacubitril/valsartan does not affect diabetes-induced increases in RRI

**Fig. 7:**
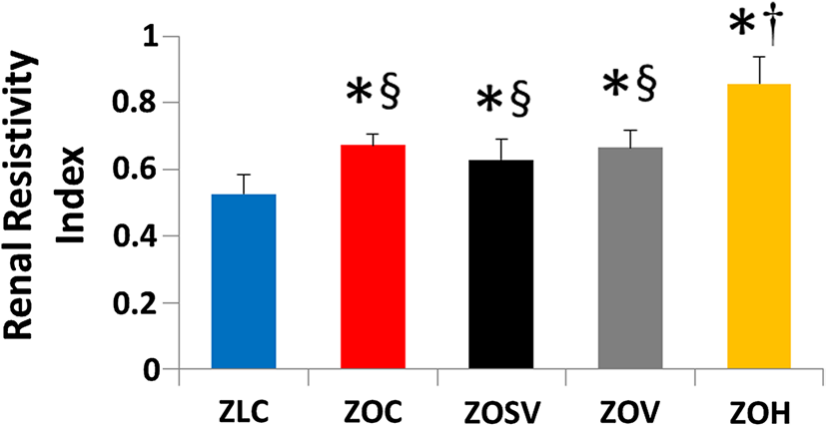
Treatment effects on the renal resistivity index. Data are represented by mean ± SE in the accompanying bar graph. n = 6–7 rats per group. Symbols: *p < 0.05 versus ZLC; ^†^p < 0.05 versus ZOC; ^§^p < 0.05 versus ZOH

Kidney microvascular resistivity is linked to reductions in eGFR, albuminuria and CKD [52–55]. Compared to ZLC, ZOC exhibited an elevation in RRI and this was not affected by either sac/val (ZOSV) or val (ZOV) (Fig. 7). Hydralazine (ZOH) tended to exacerbate RRI.

## Discussion

Sacubitril/valsartan (sac/val) was originally approved to treat HFrEF; however, a recent post hoc analysis from the PARADIGM-HF trial suggested significant improvements in GFR and CV outcomes with this drug combination, despite causing an increase in urine albumin-to-creatinine ratio [22]. Therefore, some recent pre-clinical studies have begun to explore the mechanisms underlying the renoprotective effects of this drug combination. In this regard, two recent studies demonstrated superior renoprotection with sac/val compared to val monotherapy in rat models of severe CKD due to subtotal nephrectomy [56, 57]. In the present study, we examined the effects of sac/val on glomerular, *i.e*., filtration barrier injury, as well as, tubular remodeling and kidney fibrosis, along with associated changes in biochemical parameters in ZO rats, a translationally relevant model of early stage DN. Our findings suggest that sac/val exerts similar effects on kidney fibrosis compared to val alone. However, the suppression of hyperfiltration and proteinuria due to glomerular and tubular injury appears to be well controlled in sac/val compared to val monotherapy.

We evaluated control of glycemia, BP, dyslipidemia and GFR by both sac/val and val. Not surprisingly, sac/val or val alone did not lead to significant reductions in fasting glucose or HbA1c; however, HbA1c was highest in ZOV (5.5 ± 0.5%) and lowest in ZOSV (4.4 ± 0.3; Table 1), suggesting that the addition of sacubitril to val may confer a more favorable metabolic response compared to val monotherapy in the diabetic ZO rat. Interestingly, recent clinical data demonstrated modest improvement in HbA1c levels with sac/val, but only after one (~ 0.26%) and three (~ 0.40%) years of follow up [58]. The brief duration of treatment in the present study and the fact that the principle mode of action of each drug in this combination is not known to directly regulate blood glucose, may explain why no significant improvement was observed in glycemia. Therefore, long-term studies of the efficacy of sac/val for treatment of DN are warranted. Moreover, each treatment exhibited differential effects on molecular and biochemical parameters, filtration barrier injury, GFR and glomerular and tubular remodeling, despite comparable lowering of MAP by all three treatments. This suggests that the renoprotection afforded by sac/val in this preclinical model may be independent of BP reduction.

Dyslipidemia is also known to contribute to nephropathy [59]. A previous study in male ZO rats treated with the ARB telmisartan, reported improvement in progression of DN and a tendency towards normalized plasma cholesterol and triglycerides [60]. Herein, we observed that sac/val was more effective in lowering plasma total cholesterol compared to val or hydralazine. Since hypercholesterolemia in diabetes is usually associated with increased oxidized LDL and renal injury [57, 61], it is possible that the improvement in plasma total cholesterol in ZOSV may have contributed, in part, to amelioration of kidney injury.

The observation that untreated ZO rats (ZOC) had lower plasma cystatin c indicates hyperfiltration, which is consistent with early stage DN in obese humans [3]. Our study shows a significant increase in cystatin c in sac/val treated rats indicating that the combination therapy reversed hyperfiltration. This initial reversal of hyperfiltration and reduction in GFR is consistent with inhibition of RAS activation [62], supporting the use of ARBs or ACEi in suppressing the eventual decline in GFR in diabetic patients. Recently, sodium glucose co-transporter 2 (SGLT2) inhibitors have also been shown to decrease GFR and exert renoprotection in patients with DN with baseline hyperfiltration [63, 64]. There is an apparent inconsistency between our preclinical study and results of the PARADIGM HF trial showing improvement in GFR that is likely a consequence of long treatment duration (44 months) [24]. Thus, a limitation of our study is that we only measured kidney function, i.e., GFR, during the early hyper filtrating stage of DN. We posit that a longer term treatment and follow up would likely result in protection from decline in GFR in the ZO rats treated with sac/val as observed in PARADIGM HF.

With respect to proteinuria, the magnitude of suppression of proteinuria by the combination therapy was slightly greater than val alone, suggesting that the addition of NEPi on top of an ARB elicits additional renoprotective properties. Similar to albuminuria, non-albuminuric proteinuria also reflects filtration barrier injury and glomerular dysfunction. It is also recognized as a sensitive marker of tubular injury in the setting of DN with minimal kidney injury [65]. In this study, the albuminuria was not markedly increased in ZOC, nor was it decreased in any of the treatment groups. Supporting these observations on proteinuria, the ultrastructural findings indicated healthier podocytes and their foot processes with sac/val than val alone, and this was associated with rescue of nephrin and podocin expression, suggesting that the combination therapy is more effective at improving glomerulopathy in relation to (non-albuminuric) proteinuria.

In addition to glomerulopathy, DN is characterized by tubulopathy [66]. A number of biomarkers have been validated to detect existing tubular injury or its reversal following treatment of acute kidney injury in both humans and preclinical models [67]. We observed increases in β-NAG and GTT in untreated ZO rats (ZOC) and none of the treatments affected their levels. Therefore, we examined additional sensitive biomarkers of early tubular injury. Out of eight injury biomarkers that we evaluated (Table 2), five were elevated in the untreated ZO rats with early stage DN. In this injury profile, clusterin and KIM-1 stand out as sensitive biomarkers, and their increased levels in urine indicate tubular necrosis. Of note, urinary levels of clusterin and KIM-1 have been shown to increase during early stages of DN [68–71]. Importantly, we and others have shown their suppression following treatment with ARBs [69, 72]. Moreover, sac/val has recently been shown to suppress tubular injury markers, including KIM-1, in dogs with cardiorenal syndrome [73]. Supporting these observations, our data show that sac/val suppressed both clusterin and KIM-1, while val alone suppressed only clusterin (Table 2). On the other hand, hydralazine had no significant effect on any of the markers. Thus, our data demonstrating an increase in clinically relevant tubule injury markers in diabetic rats (ZOC) and their suppression with sac/val (ZOSV), further support the renoprotective effects of sac/val. Though we have not formally identified a specific injury profile or network linked to early stage DN, nor alteration of such a construct with therapeutic intervention, other studies have highlighted the role of metabolic dysregulation as an underlying cause for progression of kidney disease, including fibrosis. This topic has recently been reviewed emphasizing the significance of a metabolomics approach in identifying molecular mechanisms contributing to CKD [74]. Future studies on metabolomics are warranted when exploring renal outcomes with sac/val treatment.

Activation of the RAS and the accompanying increase in NADPH oxidase-mediated oxidative stress is implicated in progression of DN [25, 75]. We and others have reported that ARBs are renoprotective in rat genetic [72, 76] and nutritional models of nephropathy [77]. Kidney fibrosis, comprising both periarterial and interstitial fibrosis, is also a determinant of progression of DN. In this study sac/val and val, as well as hydralazine, reduced kidney fibrosis, and this inhibition occurred in parallel with suppression of oxidative stress, AT_1_R expression and NOX2. These findings suggest that AT_1_R antagonism and reduced oxidative stress, either by use of ARBs or hemodynamic effects, might have contributed to suppression of kidney fibrosis. In this regard, we have previously reported therapeutic suppression of kidney fibrosis and improvement in filtration barrier injury in association with a decrease in oxidative stress in ZO and Ren2 rats, as evidenced by an decrease in NADPH oxidase activity and 3-NTY accumulation [27, 72, 76].

In this study, compared to ZLC, 3-NTY expression was significantly elevated in ZOC, but was markedly suppressed in the three treated groups (ZOSV, ZOV and ZOH), with associated decreases in mesangial expansion and intertubular and periarteriolar fibrosis, suggesting the role for oxidative stress in kidney fibrosis. We previously proposed that, in the setting of metabolic syndrome/diabetes, elevated tissue Ang-II induces NOX-mediated oxidative stress and loss of nephrin and podocin, leading to podocyte effacement and loss of slit pores diaphragms, and that these protein deficiencies and accompanying ultrastructural abnormalities are prerequisite to proteinuria/albuminuria [26]. In this regard, RAS inhibitors, such as ARBs and ACE inhibitors, have been reported to ameliorate loss of nephrin and podocin in the diabetic kidney of humans and rodents [48, 78].

In this study, AT_1_R, NOX2 and NOX4 gene expression and oxidative stress increased in untreated ZO rats (ZOC) and these effects occurred in concert with decreases in podocin and nephrin expression. Although AT_1_R expression and oxidative stress decreased similarly in all treatment groups, the rescue of nephrin and podocin expression occurred only in rats treated with sac/val (ZOSV), compared to val (ZOV). It is also noteworthy that sac/val was more effective at suppressing NOX4 expression compared to val and hydralazine. However, RRI, some urinary tubular injury markers and ultrastructural features were not consistently associated with treatment-related suppression in oxidative stress and fibrosis. Therefore, the renoprotective effects of sac/val may only be due, in part, to reductions in glomerular and tubular nitroso-oxidative stress. These findings suggest that other factors/pathways contribute to the efficacy of combination therapy. In this regard, recent studies have shown a role for a cGMP dependent pathway in improving podocin expression and filtration barrier [79, 80]. Natriuretic peptides act mainly through increase in cGMP thereby possibly contributing to improved renoprotection by sacubitril on filtration barrier proteins compared to val monotherapy.

High BP has been shown to contribute to proteinuria. Given that hydralazine reduced MAP, but not proteinuria, compared to sac/val, suggests that BP reduction, although important, may not be the primary determinant contributing to improvements in proteinuria by this combination. Further, mesangial expansion occurred in ZOC and was significantly reduced in ZOSV, ZOV and ZOH. However, val monotherapy (ZOV) was more effective in preventing the expansion of mesangium compared to sac/val or hydralazine. As such, treatment-related protection from mesangial expansion appears to play a lesser role in preventing glomerular injury in this model of early stage DN.

The RRI may be useful in predicting subclinical CV and kidney vascular disease in diabetes [81]. A previous study by Bruno et al. [82] reported significantly elevated RRI in patients with T2DM (0.65 ± 0.06) compared to non-diabetic individuals (0.59 ± 0.05) and patients with essential hypertension (0.58 ± 0.05). Interestingly, increased RRI in the diabetic subjects was associated with elevated albuminuria. In fact, the utility of RRI as an indicator of kidney disease was recently validated in high fat diet fed mice [83]. We recently reported increased RRI in db/db mouse, a more severe model of DN, compared to ZO, and this was associated with aortic stiffness and a reduction in renal 3-NTY [34]. In the present study, we observed elevated RRI in ZOC compared to ZLC (0.67 ± 0.02 versus 0.53 ± 0.03; p < 0.05), however, sac/val and val alone did not significantly suppress RRI. In contrast, hydralazine increased RRI, compared to ZOC (0.86 ± 0.03 versus 0.67 ± 0.02; p < 0.05), and this was associated with greater proteinuria in the ZOH group (Table 2 and Fig. 2a), suggesting a possible context-dependent association for hydralazine in contrast to sac/val and val.

## Conclusions

In summary, our study provides insights into the efficacy of sac/val combination therapy compared to val monotherapy in a preclinical model of early stage DN. Our data suggest that sac/val is slightly more renoprotective and the principal mechanism involves rescue from hyperfiltration and subsequent improvement in glomerular structure and function. However, future preclinical and clinical investigations are warranted to test whether sac/val affords renoprotection specifically in the setting of DN associated with diastolic dysfunction or HFpEF. In this regard, results of the ongoing PARAGON-HF trial (Prospective Comparison of ARNI with ARB Global Outcomes in HF with Preserved Ejection Fraction) should be forthcoming in the summer of 2019 and may offer insight into whether Entresto (sac/val) also affords renoprotection in individuals with DN associated with HFpEF. Since newer classes of antihyperglycemia drugs, including the sodium glucose cotransporter 2 (SGLT2) inhibitors, glucagon-like peptide 1 receptor (GLP1R) agonists and dipeptidyl peptidase 4 (DPP4) inhibitors, slow progression of DN [84], it remains to be determined whether combining Entresto with these newer classes of drugs could provide additional renoprotection.

## Abbreviations

ACEi: ACE inhibitor
ALT: alanine aminotransferase
AST: aspartate aminotransferase
ARB: angiotensin type 1 receptor (AT_1_R) blocker
ANP: atrial natriuretic peptide
BNP: brain natriuretic peptide
cGMP: cyclic guanosine monophosphate
CV: cardiovascular
CVD: CV disease
CKD: chronic kidney disease
DAB: diaminobenzidine
DN: diabetic nephropathy
DPP4: dipeptidyl peptidase 4
ESRD: end stage renal disease
GFR: glomerular filtration rate
eGFR: estimated GFR
GLP1R: glucagon-like peptide 1 receptor
GGT: γ-glutamyl transpeptidase
GSTα: glutathione-*S*-transferase-α
HFpEF: heart failure with preserved ejection fraction
HFrEF: heart failure with reduced ejection fraction
HOMA-IR: homeostatic model assessment of insulin resistance
IP-10: interferon-γ-inducible protein-10
KIM-1: kidney injury molecule-1
MAP: mean arterial pressure
β-NAG: *N*-acetyl-β-d-glucosaminidase
3-NTY: 3-nitrotyrosine
NEPi: neprilysin inhibition
NOX: NADPH oxidase
PARADIGM-HF trial: Prospective Comparison of ARNI with ACE inhibition to Determine Impact on Global Mortality and Morbidity in Heart Failure
PARAGON-HF trial: Prospective Comparison of ARNI with ARB Global Outcomes in HF with Preserved Ejection Fraction
PSV: peak systolic velocity
LDV: lowest diastolic velocity
OPN: osteopontin
PSR: picrosirius red
RRI: renal resistivity index
UK HARP-III: United Kingdom Heart and Renal Protection-III
sac: sacubitril
val: valsartan
sacubitril/valsartan: sac/val
RAS: renin-angiotensin system
SGLT2: sodium glucose cotransporter 2
TIMP-1: tissue inhibitor of metalloprotease
TEM: transmission electron microscopy
T2DM: type 2 diabetes mellitus
VEGF: vascular endothelial growth factor
ZLC: Zucker Lean control
ZOC: Zucker Obese control
ZOSV: Zucker Obese sac/val
ZOV: Zucker Obese valsartan
ZOH: Zucker Obese hydralazine

## References

[1] Fouli GE, Gnudi L (2018). The future: experimental therapies for renal disease in diabetes. Nephron.

[2] Flegal KM, Carroll MD, Ogden CL, Curtin LR (2010). Prevalence and trends in obesity among US adults, 1999–2008. JAMA.

[3] Whaley-Connell A, Sowers JR (2017). Obesity and kidney disease: from population to basic science and the search for new therapeutic targets. Kidney Int.

[4] Alicic RZ, Rooney MT, Tuttle KR (2017). Diabetic kidney disease: challenges, progress, and possibilities. Clin J Am Soc Nephrol.

[5] Xiao QR, Fan LJ, Jiang W, Zhao DF, Wan H, Pan DY, Lin X, Zhang T, Shen J (2016). Prevalence of chronic kidney disease and its risk factors in subjects with different glucose metabolism status. Nan Fang Yi Ke Da Xue Xue Bao.

[6] Lewis EJ, Hunsicker LG, Bain RP, Rohde RD (1993). The effect of angiotensin-converting-enzyme inhibition on diabetic nephropathy. The Collaborative Study Group. N Engl J Med.

[7] Brenner BM, Cooper ME, de Zeeuw D, Keane WF, Mitch WE, Parving HH, Remuzzi G, Snapinn SM, Zhang Z, Shahinfar S (2001). Effects of losartan on renal and cardiovascular outcomes in patients with type 2 diabetes and nephropathy. N Engl J Med.

[8] Lewis EJ, Hunsicker LG, Clarke WR, Berl T, Pohl MA, Lewis JB, Ritz E, Atkins RC, Rohde R, Raz I (2001). Renoprotective effect of the angiotensin-receptor antagonist irbesartan in patients with nephropathy due to type 2 diabetes. N Engl J Med.

[9] Ruggenenti P, Perna A, Gherardi G, Garini G, Zoccali C, Salvadori M, Scolari F, Schena FP, Remuzzi G (1999). Renoprotective properties of ACE-inhibition in non-diabetic nephropathies with non-nephrotic proteinuria. Lancet.

[10] Bayes-Genis A, Barallat J, Galan A, de Antonio M, Domingo M, Zamora E, Urrutia A, Lupon J (2015). Soluble neprilysin is predictive of cardiovascular death and heart failure hospitalization in heart failure patients. J Am Coll Cardiol.

[11] Kaplinsky E (2016). Sacubitril/valsartan in heart failure: latest evidence and place in therapy. Ther Adv Chronic Dis.

[12] Judge P, Haynes R, Landray MJ, Baigent C (2015). Neprilysin inhibition in chronic kidney disease. Nephrol Dial Transplant.

[13] Nishikimi T, Inaba-Iemura C, Ishimura K, Tadokoro K, Koshikawa S, Ishikawa K, Akimoto K, Hattori Y, Kasai K, Minamino N (2009). Natriuretic peptide/natriuretic peptide receptor-A (NPR-A) system has inhibitory effects in renal fibrosis in mice. Regul Pept.

[14] Kasahara M, Mukoyama M, Sugawara A, Makino H, Suganami T, Ogawa Y, Nakagawa M, Yahata K, Goto M, Ishibashi R (2000). Ameliorated glomerular injury in mice overexpressing brain natriuretic peptide with renal ablation. J Am Soc Nephrol.

[15] Ogawa Y, Mukoyama M, Yokoi H, Kasahara M, Mori K, Kato Y, Kuwabara T, Imamaki H, Kawanishi T, Koga K (2012). Natriuretic peptide receptor guanylyl cyclase-A protects podocytes from aldosterone-induced glomerular injury. J Am Soc Nephrol.

[16] Bohm M, Young R, Jhund PS, Solomon SD, Gong J, Lefkowitz MP, Rizkala AR, Rouleau JL, Shi VC, Swedberg K (2017). Systolic blood pressure, cardiovascular outcomes and efficacy and safety of sacubitril/valsartan (LCZ696) in patients with chronic heart failure and reduced ejection fraction: results from PARADIGM-HF. Eur Heart J.

[17] von Lueder TG, Wang BH, Kompa AR, Huang L, Webb R, Jordaan P, Atar D, Krum H (2015). Angiotensin receptor neprilysin inhibitor LCZ696 attenuates cardiac remodeling and dysfunction after myocardial infarction by reducing cardiac fibrosis and hypertrophy. Circ Heart Fail.

[18] Gori M, Senni M (2016). Sacubitril/valsartan (LCZ696) for the treatment of heart failure. Expert Rev Cardiovasc Ther.

[19] Jhund PS, Fu M, Bayram E, Chen CH, Negrusz-Kawecka M, Rosenthal A, Desai AS, Lefkowitz MP, Rizkala AR, Rouleau JL (2015). Efficacy and safety of LCZ696 (sacubitril–valsartan) according to age: insights from PARADIGM-HF. Eur Heart J.

[20] Okumura N, Jhund PS, Gong J, Lefkowitz MP, Rizkala AR, Rouleau JL, Shi VC, Swedberg K, Zile MR, Solomon SD (2016). Effects of sacubitril/valsartan in the PARADIGM-HF trial (prospective comparison of ARNI with ACEI to determine impact on global mortality and morbidity in heart failure) according to background therapy. Circ Heart Fail.

[21] Hubers SA, Brown NJ (2016). Combined angiotensin receptor antagonism and neprilysin inhibition. Circulation.

[22] Damman K, Gori M, Claggett B, Jhund PS, Senni M, Lefkowitz MP, Prescott MF, Shi VC, Rouleau JL, Swedberg K (2018). Renal effects and associated outcomes during angiotensin–neprilysin inhibition in heart failure. JACC Heart Fail.

[23] Haynes R, Judge PK, Staplin N, Herrington WG, Storey BC, Bethel A, Bowman L, Brunskill N, Cockwell P, Hill M (2018). Effects of sacubitril/valsartan versus irbesartan in patients with chronic kidney disease. Circulation.

[24] Packer M, Claggett B, Lefkowitz MP, McMurray JJV, Rouleau JL, Solomon SD, Zile MR (2018). Effect of neprilysin inhibition on renal function in patients with type 2 diabetes and chronic heart failure who are receiving target doses of inhibitors of the renin-angiotensin system: a secondary analysis of the PARADIGM-HF trial. Lancet Diabetes Endocrinol.

[25] Whaley-Connell A, Pulakat L, Demarco VG, Hayden MR, Habibi J, Henriksen EJ, Sowers JR (2011). Overnutrition and the cardiorenal syndrome: use of a rodent model to examine mechanisms. Cardiorenal Med.

[26] Whaley-Connell A, DeMarco VG, Lastra G, Manrique C, Nistala R, Cooper SA, Westerly B, Hayden MR, Wiedmeyer C, Wei Y (2008). Insulin resistance, oxidative stress, and podocyte injury: role of rosuvastatin modulation of filtration barrier injury. Am J Nephrol.

[27] Nistala R, Habibi J, Aroor A, Sowers JR, Hayden MR, Meuth A, Knight W, Hancock T, Klein T, DeMarco VG (2014). DPP4 inhibition attenuates filtration barrier injury and oxidant stress in the Zucker Obese rat. Obesity (Silver Spring).

[28] Hayashi K, Kanda T, Homma K, Tokuyama H, Okubo K, Takamatsu I, Tatematsu S, Kumagai H, Saruta T (2002). Altered renal microvascular response in Zucker Obese rats. Metabolism.

[29] Park SK, Kang SK (1995). Renal function and hemodynamic study in obese Zucker rats. Korean J Intern Med.

[30] Aroor AR, Sowers JR, Bender SB, Nistala R, Garro M, Mugerfeld I, Hayden MR, Johnson MS, Salam M, Whaley-Connell A (2013). Dipeptidylpeptidase Inhibition is associated with improvement in blood pressure and diastolic function in insulin resistant male Zucker Obese rats. Endocrinology.

[31] Zhou X, Ma L, Habibi J, Whaley-Connell A, Hayden MR, Tilmon RD, Brown AN, Kim JA, Demarco VG, Sowers JR (2010). Nebivolol improves diastolic dysfunction and myocardial remodeling through reductions in oxidative stress in the Zucker Obese rat. Hypertension.

[32] Bender SB, DeMarco VG, Padilla J, Jenkins NT, Habibi J, Garro M, Pulakat L, Aroor AR, Jaffe IZ, Sowers JR (2015). Mineralocorticoid receptor antagonism treats obesity-associated cardiac diastolic dysfunction. Hypertension.

[33] DeVallance E, Branyan KW, Lemaster K, Olfert IM, Smith DM, Pistilli EE, Frisbee JC, Chantler PD (2018). Aortic dysfunction in metabolic syndrome mediated by perivascular adipose tissue TNFalpha- and NOX2-dependent pathway. Exp Physiol.

[34] Aroor AR, Das NA, Carpenter AJ, Habibi J, Jia G, Ramirez-Perez FI, Martinez-Lemus L, Manrique-Acevedo CM, Hayden MR, Duta C (2018). Glycemic control by the SGLT2 inhibitor empagliflozin decreases aortic stiffness, renal resistivity index and kidney injury. Cardiovasc Diabetol.

[35] Pofi R, Fiore D, De Gaetano R, Panio G, Gianfrilli D, Pozza C, Barbagallo F, Xiang YK, Giannakakis K, Morano S (2017). Phosphodiesterase-5 inhibition preserves renal hemodynamics and function in mice with diabetic kidney disease by modulating miR-22 and BMP7. Sci Rep.

[36] Westergren HU, Gronros J, Heinonen SE, Miliotis T, Jennbacken K, Sabirsh A, Ericsson A, Jonsson-Rylander AC, Svedlund S, Gan LM (2015). Impaired coronary and renal vascular function in spontaneously type 2 diabetic leptin-deficient mice. PLoS ONE.

[37] Di Lascio N, Kusmic C, Stea F, Lenzarini F, Barsanti C, Leloup A, Faita F (2018). Longitudinal micro-ultrasound assessment of the ob/ob mouse model: evaluation of cardiovascular, renal and hepatic parameters. Int J Obes (Lond).

[38] Kawai T, Kamide K, Onishi M, Yamamoto-Hanasaki H, Baba Y, Hongyo K, Shimaoka I, Tatara Y, Takeya Y, Ohishi M (2011). Usefulness of the resistive index in renal Doppler ultrasonography as an indicator of vascular damage in patients with risks of atherosclerosis. Nephrol Dial Transplant.

[39] Habibi J, Aroor AR, Sowers JR, Jia G, Hayden MR, Garro M, Barron B, Mayoux E, Rector RS, Whaley-Connell A (2017). Sodium glucose transporter 2 (SGLT2) inhibition with empagliflozin improves cardiac diastolic function in a female rodent model of diabetes. Cardiovasc Diabetol.

[40] Habibi J, Hayden MR, Sowers JR, Pulakat L, Tilmon RD, Manrique C, Lastra G, Demarco VG, Whaley-Connell A (2011). Nebivolol attenuates redox-sensitive glomerular and tubular mediated proteinuria in obese rats. Endocrinology.

[41] Aroor AR, Jia G, Habibi J, Sun Z, Ramirez-Perez FI, Brady B, Chen D, Martinez-Lemus LA, Manrique C, Nistala R (2017). Uric acid promotes vascular stiffness, maladaptive inflammatory responses and proteinuria in western diet fed mice. Metabolism.

[42] Padilla J, Carpenter AJ, Das NA, Kandikattu HK, Lopez-Ongil S, Martinez-Lemus LA, Siebenlist U, DeMarco VG, Chandrasekar B (2018). TRAF3IP2 mediates high glucose-induced endothelin-1 production as well as endothelin-1-induced inflammation in endothelial cells. Am J Physiol Heart Circ Physiol.

[43] Yariswamy M, Yoshida T, Valente AJ, Kandikattu HK, Sakamuri SS, Siddesha JM, Sukhanov S, Saifudeen Z, Ma L, Siebenlist U (2016). Cardiac-restricted overexpression of TRAF3 interacting protein 2 (TRAF3IP2) results in spontaneous development of myocardial hypertrophy, fibrosis, and dysfunction. J Biol Chem.

[44] Valente AJ, Irimpen AM, Siebenlist U, Chandrasekar B (2014). OxLDL induces endothelial dysfunction and death via TRAF3IP2: inhibition by HDL3 and AMPK activators. Free Radic Biol Med.

[45] Hermida J, Romero R, Tutor JC (2002). Relationship between serum cystatin C and creatinine in kidney and liver transplant patients. Clin Chim Acta.

[46] Suzuki H, Yamamoto T, Fujigaki Y, Eguchi S, Hishida A (2011). Comparison of ROCK and EGFR activation pathways in the progression of glomerular injuries in AngII-infused rats. Ren Fail.

[47] Jim B, Ghanta M, Qipo A, Fan Y, Chuang PY, Cohen HW, Abadi M, Thomas DB, He JC (2012). Dysregulated nephrin in diabetic nephropathy of type 2 diabetes: a cross sectional study. PLoS ONE.

[48] Bonnet F, Cooper ME, Kawachi H, Allen TJ, Boner G, Cao Z (2001). Irbesartan normalises the deficiency in glomerular nephrin expression in a model of diabetes and hypertension. Diabetologia.

[49] Doublier S, Salvidio G, Lupia E, Ruotsalainen V, Verzola D, Deferrari G, Camussi G (2003). Nephrin expression is reduced in human diabetic nephropathy: evidence for a distinct role for glycated albumin and angiotensin II. Diabetes.

[50] Nistala R, Whaley-Connell A (2013). Resistance to insulin and kidney disease in the cardiorenal metabolic syndrome; role for angiotensin II. Mol Cell Endocrinol.

[51] Sedeek M, Nasrallah R, Touyz RM, Hebert RL (2013). NADPH oxidases, reactive oxygen species, and the kidney: friend and foe. J Am Soc Nephrol.

[52] Zhang J, Bottiglieri T, McCullough PA (2017). The central role of endothelial dysfunction in cardiorenal syndrome. Cardiorenal Med.

[53] Woodard T, Sigurdsson S, Gotal JD, Torjesen AA, Inker LA, Aspelund T, Eiriksdottir G, Gudnason V, Harris TB, Launer LJ (2015). Mediation analysis of aortic stiffness and renal microvascular function. J Am Soc Nephrol.

[54] Calabia J, Torguet P, Garcia I, Martin N, Mate G, Marin A, Molina C, Valles M (2014). The relationship between renal resistive index, arterial stiffness, and atherosclerotic burden: the link between macrocirculation and microcirculation. J Clin Hypertens (Greenwich).

[55] Mitchell GF (2008). Effects of central arterial aging on the structure and function of the peripheral vasculature: implications for end-organ damage. J Appl Physiol (1985).

[56] Jing W, Vaziri ND, Nunes A, Suematsu Y, Farzaneh T, Khazaeli M, Moradi H (2017). LCZ696 (sacubitril/valsartan) ameliorates oxidative stress, inflammation, fibrosis and improves renal function beyond angiotensin receptor blockade in CKD. Am J Transl Res.

[57] Ushijima K, Ando H, Arakawa Y, Aizawa K, Suzuki C, Shimada K, Tsuruoka SI, Fujimura A (2017). Prevention against renal damage in rats with subtotal nephrectomy by sacubitril/valsartan (LCZ696), a dual-acting angiotensin receptor-neprilysin inhibitor. Pharmacol Res Perspect.

[58] Seferovic JP, Claggett B, Seidelmann SB, Seely EW, Packer M, Zile MR, Rouleau JL, Swedberg K, Lefkowitz M, Shi VC (2017). Effect of sacubitril/valsartan versus enalapril on glycaemic control in patients with heart failure and diabetes: a post hoc analysis from the PARADIGM-HF trial. Lancet Diabetes Endocrinol.

[59] Dominguez JH, Tang N, Xu W, Evan AP, Siakotos AN, Agarwal R, Walsh J, Deeg M, Pratt JH, March KL (2000). Studies of renal injury III: lipid-induced nephropathy in type II diabetes. Kidney Int.

[60] Ohmura T, Tsunenari I, Seidler R, Chachin M, Hayashi T, Konomi A, Matsumaru T, Sumida T, Hayashi N, Horie Y (2012). Renoprotective effects of telmisartan on renal injury in obese Zucker rats. Acta Diabetol.

[61] Furukawa S, Suzuki H, Fujihara K, Kobayashi K, Iwasaki H, Sugano Y, Yatoh S, Sekiya M, Yahagi N, Shimano H (2018). Malondialdehyde-modified LDL-related variables are associated with diabetic kidney disease in type 2 diabetes. Diabetes Res Clin Pract.

[62] Palatini P (2012). Glomerular hyperfiltration: a marker of early renal damage in pre-diabetes and pre-hypertension. Nephrol Dial Transplant.

[63] Barnett AH, Mithal A, Manassie J, Jones R, Rattunde H, Woerle HJ, Broedl UC (2014). investigators E-RRt: efficacy and safety of empagliflozin added to existing antidiabetes treatment in patients with type 2 diabetes and chronic kidney disease: a randomised, double-blind, placebo-controlled trial. Lancet Diabetes Endocrinol.

[64] Novikov A, Vallon V (2016). Sodium glucose cotransporter 2 inhibition in the diabetic kidney: an update. Curr Opin Nephrol Hypertens.

[65] Kim SS, Song SH, Kim IJ, Kim WJ, Jeon YK, Kim BH, Kwak IS, Lee EK, Kim YK (2014). Nonalbuminuric proteinuria as a biomarker for tubular damage in early development of nephropathy with type 2 diabetic patients. Diabetes Metab Res Rev.

[66] Gilbert RE, Cooper ME (1999). The tubulointerstitium in progressive diabetic kidney disease: more than an aftermath of glomerular injury?. Kidney Int.

[67] Alge JL, Arthur JM (2015). Biomarkers of AKI: a review of mechanistic relevance and potential therapeutic implications. Clin J Am Soc Nephrol.

[68] Fiseha T, Tamir Z (2016). Urinary markers of tubular injury in early diabetic nephropathy. Int J Nephrol.

[69] Tuncdemir M, Ozturk M (2008). The effects of ACE inhibitor and angiotensin receptor blocker on clusterin and apoptosis in the kidney tissue of streptozotocin-diabetic rats. J Mol Histol.

[70] Korrapati MC, Shaner BE, Neely BA, Alge JL, Arthur JM, Schnellmann RG (2012). Diabetes-induced renal injury in rats is attenuated by suramin. J Pharmacol Exp Ther.

[71] Chaudhary K, Phadke G, Nistala R, Weidmeyer CE, McFarlane SI, Whaley-Connell A (2010). The emerging role of biomarkers in diabetic and hypertensive chronic kidney disease. Curr Diab Rep.

[72] Whaley-Connell A, Habibi J, Panfili Z, Hayden MR, Bagree S, Nistala R, Hyder S, Krueger B, Demarco V, Pulakat L (2011). Angiotensin II activation of mTOR results in tubulointerstitial fibrosis through loss of N-cadherin. Am J Nephrol.

[73] Sabbah HN, Zhang K, Zu J, Gupta R, Singh-Gupta V (2017). Therapy with saculbitril/valsartan improves left ventricular systolic function and biomarkers of kidney injury in dogs with experimentally-induced cardiorenal syndrome. Circulation.

[74] Hocher B, Adamski J (2017). Metabolomics for clinical use and research in chronic kidney disease. Nat Rev Nephrol.

[75] Lv W, Booz GW, Fan F, Wang Y, Roman RJ (2018). Oxidative stress and renal fibrosis: recent insights for the development of novel therapeutic strategies. Front Physiol.

[76] Whaley-Connell A, Chowdhury N, Hayden MR, Stump CS, Habibi J, Wiedmeyer CE, Gallagher PE, Tallant EA, Cooper SA, Link CD (2006). Oxidative stress and glomerular filtration barrier injury: role of the renin-angiotensin system in the Ren2 transgenic rat. Am J Physiol Renal Physiol.

[77] Dai HY, Zheng M, Tang RN, Ma KL, Ni J, Liu BC (2012). Inhibition of integrin-linked kinase by angiotensin II receptor antagonist, irbesartan attenuates podocyte injury in diabetic rats. Chin Med J (Engl).

[78] Langham RG, Kelly DJ, Cox AJ, Thomson NM, Holthofer H, Zaoui P, Pinel N, Cordonnier DJ, Gilbert RE (2002). Proteinuria and the expression of the podocyte slit diaphragm protein, nephrin, in diabetic nephropathy: effects of angiotensin converting enzyme inhibition. Diabetologia.

[79] Krishnan SM, Kraehling JR, Eitner F, Benardeau A, Sandner P (2018). The impact of the nitric oxide (NO)/soluble guanylyl cyclase (sGC) signaling cascade on kidney health and disease: a preclinical perspective. Int J Mol Sci.

[80] Fang L, Radovits T, Szabo G, Mozes MM, Rosivall L, Kokeny G (2013). Selective phosphodiesterase-5 (PDE-5) inhibitor vardenafil ameliorates renal damage in type 1 diabetic rats by restoring cyclic 3′,5′ guanosine monophosphate (cGMP) level in podocytes. Nephrol Dial Transplant.

[81] Lubas A, Kade G, Niemczyk S (2014). Renal resistive index as a marker of vascular damage in cardiovascular diseases. Int Urol Nephrol.

[82] Bruno RM, Daghini E, Landini L, Versari D, Salvati A, Santini E, Di Paco I, Magagna A, Taddei S, Ghiadoni L (2011). Dynamic evaluation of renal resistive index in normoalbuminuric patients with newly diagnosed hypertension or type 2 diabetes. Diabetologia.

[83] Xu H, Ma Z, Lu S, Li R, Lyu L, Ding L, Lu Q (2017). Renal resistive index as a novel indicator for renal complications in high-fat diet-fed mice. Kidney Blood Press Res.

[84] Hocher B, Tsuprykov O (2017). Diabetic nephropathy: renoprotective effects of GLP1R agonists and SGLT2 inhibitors. Nat Rev Nephrol.

